# Association between fundus tessellated density and subfoveal choroidal thickness in children with different refractive statuses

**DOI:** 10.3389/fmed.2026.1713592

**Published:** 2026-03-05

**Authors:** Cong Zhang, Yue Yan, Lingli Zhang, Jie Wang, Xingye Wang, Xiyuan Zhou

**Affiliations:** 1 Department of Ophthalmology, Affiliated Second Hospital, Chongqing Medical University, Chongqing, China; 2 Department of Ophthalmology, Affiliated Shapingba Hospital, Chongqing University, Chongqing, China; 3 EVision Technology (Beijing) Co. Ltd., Beijing, China

**Keywords:** artificial intelligence, biomarker, fundus tessellated density, pediatric myopia, subfoveal choroidal thickness

## Abstract

**Objective:**

Present study aimed to quantitatively evaluate the association between fundus tessellated density (FTD) and subfoveal choroidal thickness (SFCT) in children with different refractive statuses and to explore the potential of FTD as a non-invasive biomarker for monitoring myopic progression.

**Methods:**

A cross-sectional study was conducted involving 619 eyes of 315 children aged 6–12 years. Participants were classified into four refractive groups: hyperopia, pre-myopia, low myopia, and moderate-to-high myopia. FTD was quantitatively assessed using artificial intelligence (AI)-based analysis of color fundus photographs. SFCT and central subfield thickness (CST) were measured using swept-source optical coherence tomography (SS-OCT). Correlation and threshold effect analyses were performed to examine the relationship between FTD and SFCT.

**Results:**

SFCT decreased significantly with increasing myopic severity (*p* < 0.001), while FTD showed a corresponding increase (*p* < 0.001). A significant negative correlation was observed between FTD and SFCT in pre-myopia (*r* = −0.183, *p* = 0.019), low myopia (*r* = −0.335, *p* < 0.001), and moderate-to-high myopia (*r* = −0.222, *p* = 0.008) groups, but not in hyperopia (*p* = 0.454). A nonlinear threshold effect was identified: when SFCT decreased below 148.90 μm in the overall cohort, FTD increased markedly, suggesting that this value may serve as a potential reference point for myopia management. This threshold decreased with higher myopic severity. CST did not follow a linear gradient with refraction but was increased in moderate-to-high myopia.

**Conclusion:**

FTD and SFCT are significantly associated in a nonlinear manner in myopic children, with a defined threshold effect that varies by refractive status. AI-based FTD measurement offers a reproducible and accessible method for quantifying early structural changes in myopia. The identified nonlinear relationship and threshold suggest that combined assessment of FTD and SFCT may hold promise for improving early detection and monitoring of pediatric myopic progression, warranting further longitudinal validation.

## Introduction

The global prevalence of myopia has been increasing dramatically, with the rate among adolescents in China being among the highest worldwide (1). In recent years, the trend of earlier onset and greater severity of myopia has become increasingly prominent (2), significantly raising the risk of vision impairment due to myopic complications and placing a substantial burden on families and society (3). Therefore, identifying reliable biomarkers that can signal rapid myopic progression is crucial for enabling early follow-up and intervention in high-risk individuals (3). From a clinical perspective, the progression from simple myopia to pathological myopia is invariably accompanied by structural changes in the eyeball and fundus morphology. The international classification system for myopic maculopathy (MMD), proposed by Ohno-Matsui et al. (4), defines tessellated fundus as the earliest lesion. This condition may remain stable for extended periods or progress to macular atrophy (5). Studies have shown that children and adolescents who develop tessellated fundus at an early stage are more likely to experience severe progression of myopic chorioretinopathy (6). Thus, accurate characterization of these fundus changes is essential for preventing further retinal and choroidal degeneration (7).

The progression of MMD is closely associated with spherical equivalent (SE), SFCT, and axial length (AL) (8). Eyes with an earlier onset of tessellated fundus typically exhibit a thinner choroid (9), indicating a strong relationship between tessellated fundus and choroidal thickness. However, previous studies have largely relied on qualitative or semi-quantitative assessments, limiting the precise quantification of this association. Recent advances in artificial intelligence (AI)-based image analysis have led to the introduction of FTD as an objective and quantitative measure, defined as the proportion of exposed choroidal area per unit area in the fundus image (10–14). Several studies have automated FTD measurement using AI algorithms involving image preprocessing, region annotation, semantic segmentation, and density calculation, significantly improving the standardization and reproducibility of evaluations (11, 15).

Evidence indicates a significant negative correlation between FTD and SFCT. For instance, large-scale population-based studies have demonstrated that higher grades of tessellated fundus are associated with significantly reduced SFCT, independent of traditional myopic parameters such as AL and SE (16, 17). Furthermore, FTD has been established as a reliable biomarker for early myopic maculopathy, with a validated cutoff value (e.g., FTD ≥ 2.22%) derived from ROC curve analysis, demonstrating good diagnostic performance (AUC = 0.774) (18). Prospective studies have further suggested that higher baseline FTD in children predicts future elongation of AL and progression of refractive error (19), highlighting its potential as a predictor of myopia progression.

Therefore, this study aims to quantitatively evaluate the correlation between FTD and SFCT in a pediatric myopic population using AI technology, compare these metrics across subgroups with varying degrees of myopia, and explore the clinical value of combined FTD and SFCT assessment as non-invasive biomarkers for monitoring myopia progression and guiding early intervention.

## Materials and methods

### Study population

This cross-sectional observational study recruited children and adolescents aged 6–12 years from the Ophthalmology Department of Shapingba Hospital Affiliated to Chongqing University between January 2024 and July 2025. A total of 619 eyes were included. Myopia was defined as a cycloplegic SE ≤ −0.50 D. Inclusion criteria comprised: (1) age 6–12 years; (2) best-corrected visual acuity (BCVA) ≥ 1.0 in both eyes, with astigmatism ≤ − 2.00 D; (3) transparent refractive media, determined by an optical coherence tomography (OCT) quality score ≥ 7/10; and (4) normal intraocular pressure and absence of strabismus, amblyopia, or other organic ocular diseases. Exclusion criteria were: (1) history of chorioretinal or vitreoretinal diseases; (2) other ocular or systemic comorbidities; (3) previous ocular surgery or trauma, or media opacities affecting imaging quality; (4) use of any ocular medication or myopia control treatment (other than spectacles) within the past 3 months; and (5) inability to cooperate with ophthalmic examinations. Based on cycloplegic SE, participants were classified into four refractive groups: hyperopia (SE > +0.75 D), pre-myopia (+0.75 D ≥ SE > −0.50 D), low myopia (−3.00 D ≤ SE ≤ −0.50 D), and moderate-to-high myopia (SE < −3.00 D). The study adhered to the ethical principles of the Declaration of Helsinki and was approved by the Ethics Committee of Shapingba Hospital Affiliated to Chongqing University (permit no. KY202424). Written informed consent was provided by all parents or legal guardians, and verbal assent was obtained from each child before examination.

### Ophthalmic examination

All participants underwent a comprehensive ophthalmic examination. The protocol included: (1) anterior segment assessment via slit-lamp biomicroscopy and fundus evaluation using direct ophthalmoscopy; (2) intraocular pressure (IOP) measurement with a non-contact tonometer (TX-20, Canon, Japan); (3) cycloplegic autorefraction performed 30 min after administering compound tropicamide eye drops (Zhuobi’an, Shenyang Xingqi Pharmaceutical Co., Ltd., China) three times at 10-min intervals. Adequate cycloplegia was confirmed by the absence of pupillary light reflex and a measured pupil diameter exceeding 6 mm. Cycloplegia was confirmed by the absence of pupillary light reflex and a pupil diameter exceeding 6 mm. Static refraction was measured by a trained optometrist; three readings were taken and averaged. Measurements were repeated if any two values differed by more than 0.50 D. Spherical equivalent was calculated as sphere power plus half cylinder power; (4) axial length (AL) measurement using the IOLmaster 700 (Carl Zeiss, USA).

### Central subfield thickness (CST) measurement

Macular region imaging was conducted using a swept-source optical coherence tomography (SS-OCT) system (DRI OCT Triton, Topcon, Japan) with a 512 × 128 scan protocol. All OCT scans were performed between 1:00 p.m. and 5:00 p.m. to minimize diurnal variation in measurements. The integrated software automatically segmented the macular area into nine subfields based on the standardized Early Treatment Diabetic Retinopathy Study (ETDRS) grid. All retinal thickness measurements were automatically adjusted for ocular magnification using the device’s built-in algorithm, which incorporates the individual’s axial length. Two experienced ophthalmologists performed all measurements independently, and three consecutive readings were obtained for each subject and averaged to improve measurement reliability and reduce inter-scan variability. CST was defined as the mean retinal thickness within a 1-mm diameter circular zone centered on the foveal depression, and this value was used for statistical analysis. As a quantitative indicator of central macular thickness, CST enables objective assessment of pathological conditions such as macular edema or retinal atrophy.

### Assessment of subfoveal choroidal thickness (SFCT)

**Figure 1:**
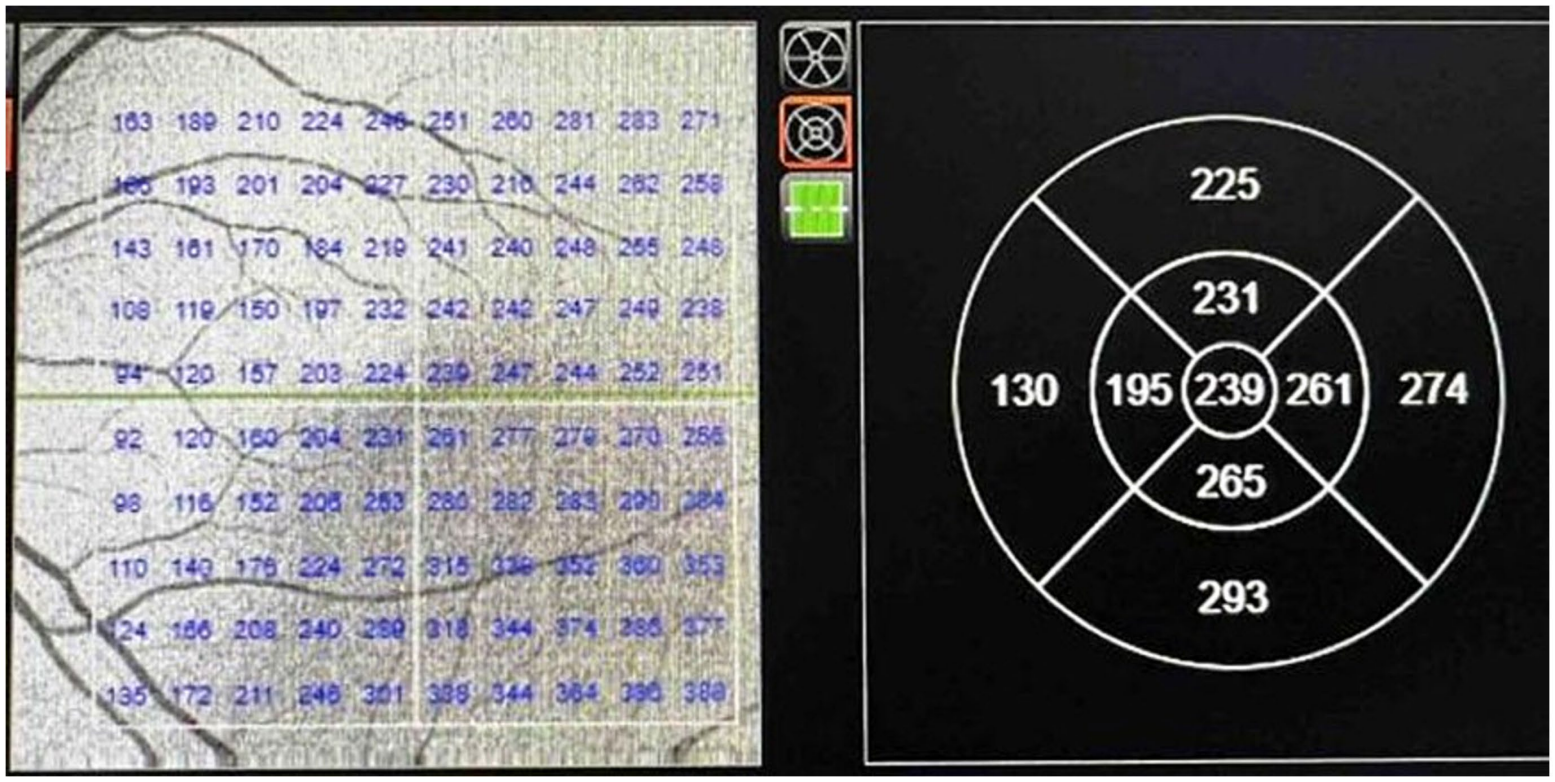
Quantitative map of subfoveal choroidal thickness in the macular region.

SFCT was measured using the enhanced-depth imaging (EDI) technique on a swept-source optical coherence tomography (SS-OCT) system (DRI OCT Triton, Topcon, Japan). This examination was also conducted within the standardized afternoon time window (1:00 p.m.–5:00 p.m.). This method improves the visualization of the choroid-scleral interface by optimizing depth penetration and signal quality. SFCT was defined as the perpendicular distance from the hyperreflective line of Bruch’s membrane to the choroidal-scleral junction at the foveal center. To enhance measurement reliability, the mean choroidal thickness within a 1-mm diameter circular area centered on the fovea was calculated and used for statistical analysis (Figure 1). The reported choroidal thickness values have been corrected for ocular magnification based on the subject’s axial length. All measurements were performed by two masked, experienced examiners, with disagreements resolved by consensus or a third senior retinal specialist.

### Quantification of fundus tessellated density (FTD)

Wide-field fundus photography was performed using a non-mydriatic camera (CLARUS 500, Carl Zeiss, Germany) to capture true-color images centered on the macula. Quantitative assessment of FTD was conducted using intelligent fundus image analysis software (EVisionAI, EVision Technology (Beijing) Co. Ltd.) (17). In accordance with established methodologies, a deep learning-based artificial intelligence image processing system was employed to extract areas of exposed choroid from the fundus images (16). FTD was defined as the percentage of the exposed choroidal area relative to the total area of a standardized region of interest (ROI). The ROI was a circular area with a diameter of 6 mm centered on the macula, from which the optic disc and major vascular arcades were automatically excluded. FTD was calculated using the formula: FTD = Area of Exposed Choroid within ROI / Total Area of ROI. The computational process consisted of four main stages: image preprocessing, sample annotation, deep learning-based segmentation, and FTD calculation. Specifically, each image underwent preprocessing—including region of interest extraction, noise reduction, normalization, and enhancement—to standardize image quality and improve feature visibility (11, 15). Annotation was performed via a semi-automated approach combining initial AI-generated labels with manual refinement by trained graders. A semantic segmentation model based on a fully convolutional network with a ResNet backbone (ResNet-FCN) was utilized to precisely delineate the exposed choroidal regions (20). The AI model (TransUnet architecture within EVisionAI) was developed and validated on large-scale, multi-center datasets, including the Beijing Eye Study cohort (11). Its performance for fundus tessellation segmentation has been rigorously evaluated. As reported in independent validation studies, the system achieved an Area Under the Curve (AUC) > 0.98 for detecting severe fundus tessellation, with sensitivity and specificity exceeding 94% (15). Additionally, related segmentation modules (e.g., for optic disc and vessels) demonstrated high accuracy, with Intersection over Union (IoU) values of 0.939 and >0.99, respectively (11, 20). These established metrics confirm the robustness and reliability of the AI-derived FTD measurements used in this study. Finally, the overall FTD was computed for each fundus image as the average exposed choroidal area per unit area of the fundus.

### Statistical analysis

Data analysis was performed using SPSS version 27.0 (IBM Corp., Chicago, IL, USA). To account for the within-subject correlation from including both eyes, key analyses involving SFCT and FTD were performed using a generalized estimating equation (GEE) model with an exchangeable working correlation structure. The linear model specified SFCT as the dependent variable, refractive status as a fixed factor, and both FTD and age as covariates, with subject ID defined as the subject variable to appropriately cluster observations within each individual. Continuous variables with normal distribution are expressed as mean ± standard deviation, while non-normally distributed data are summarized as medians with interquartile ranges (IQRs). Categorical variables are presented as frequencies and percentages. Group comparisons were conducted using one-way analysis of variance (ANOVA) with Bonferroni post-hoc test for normally distributed continuous variables, the Kruskal-Wallis test for non-normally distributed variables, and the chi-square test or Fisher’s exact test for categorical variables, as appropriate. Correlations between variables were assessed using Pearson correlation analysis for normally distributed continuous variables and Spearman’s rank correlation for ordinal or non-normally distributed data. The non-linear association between SFCT and FTD was analyzed using the XY analytics module in GraphPad Prism 8 (GraphPad Software, San Diego, CA). A cubic spline curve was fitted to capture complex non-linear relationships, and a locally weighted scatterplot smoothing (LOWESS) regression was subsequently applied with a smoothing fraction of [0.5] to visualize underlying data trends without imposing parametric assumptions. A *p*-value < 0.05 was considered statistically significant.

## Results

### Demographic and ocular characteristics

A total of 315 children were initially enrolled in this study, with both eyes of each participant examined. After excluding 11 eyes for reasons including overexposure resulting in ungradable fundus images (5 eyes), astigmatism of −2.00 D or worse (4 eyes), and best-corrected visual acuity (BCVA) less than 1.0 (2 eyes), the final analysis included 619 eyes. These eyes were categorized into four refractive groups: hyperopia (156 eyes), pre-myopia (164 eyes), low myopia (158 eyes), and moderate-to-high myopia (141 eyes).

The demographic and ocular characteristics of the participants, stratified by refractive group, are summarized in Table 1. There were no statistically significant differences in number (*p* = 0.603), age (*p* = 0.094) or sex distribution (*p* = 0.542) among the groups. However, significant differences were observed in spherical equivalent (SE), axial length (AL), and central subfield thickness (CST) (all *p* < 0.001).

**Table 1:** Baseline characteristics of study participants by refractive status.

Characteristic	Hyperopia group	Pre-myopia group	Low myopia group	Moderate-to-high myopia group	*p* value
Number of eyes	156	164	158	141	0.603
Age (years), Mean ± SD	9.62 ± 1.51	9.65 ± 1.73	9.90 ± 1.50	9.99 ± 1.58	0.094
Sex (Male/Female)	73/83	81/83	72/86	58/83	0.542
SE (D), Median (IQR)	1.25 (1.00,1.75)	0 (−0.25,0)	−1.25 (−1.66,−1.00)	−3.63 (−4.34,−3.25)	<0.001
AL (mm), Mean ± SD	22.77 ± 0.99	23.63 ± 0.81	24.09 ± 0.77	24.96 ± 0.91	<0.001
CST (μm), Mean ± SD	220.16 ± 16.88	226.12 ± 18.76	222.66 ± 16.59	230.89 ± 18.74	<0.001

The median SE values were 1.25 D (IQR: 1.00, 1.75) in the hyperopia group, 0.00 D (IQR: −0.25, 0.00) in the pre-myopia group, −1.25 D (IQR: −1.66, −1.00) in the low myopia group, and −3.63 D (IQR: −4.34, −3.25) in the moderate-to-high myopia group. Axial length also differed significantly among groups (*p* < 0.001), showing a progressive increase with myopic severity: 22.77 ± 0.99 mm in the hyperopia group, 23.63 ± 0.81 mm in the pre-myopia group, 24.09 ± 0.77 mm in the low myopia group, and 24.96 ± 0.91 mm in the moderate-to-high myopia group. Similarly, CST values varied significantly (*p* < 0.001), with the highest mean values observed in the moderate-to-high myopia group (230.89 ± 18.74 μm) and the lowest in the hyperopia group (220.16 ± 16.88 μm).

### Differences in SFCT and FTD across refractive groups

**Figure 2:**
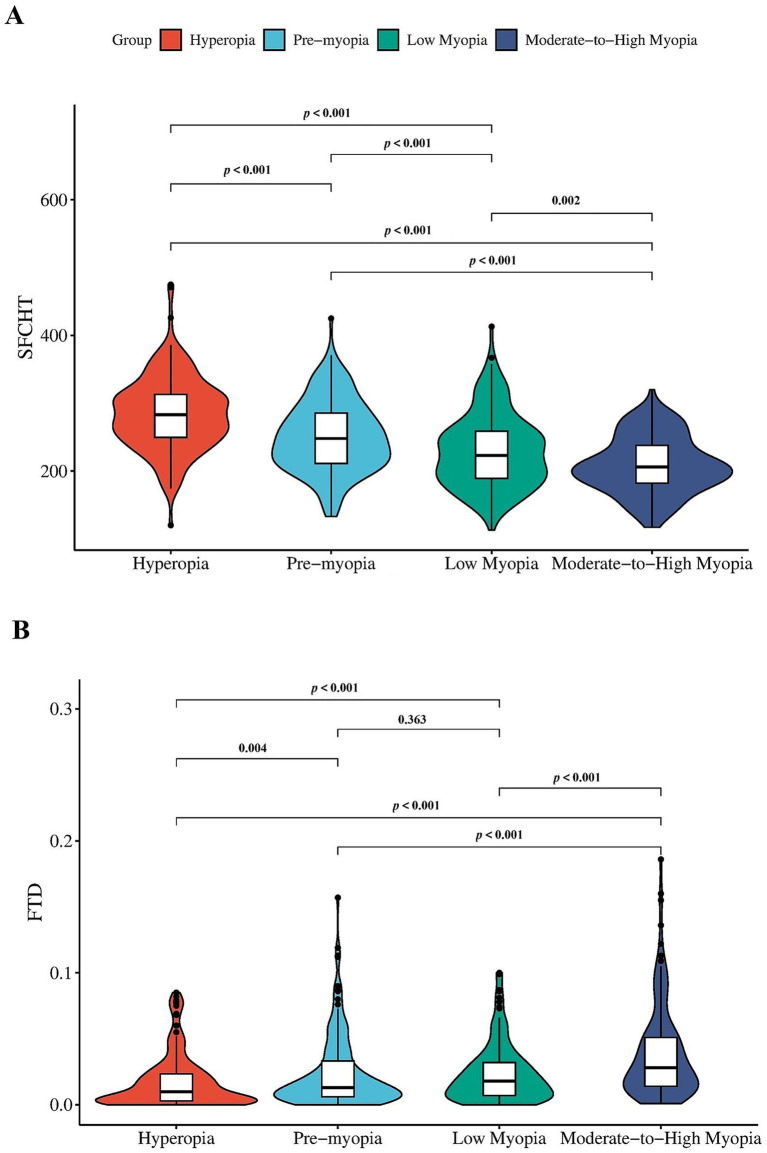
Comparison of SFCT and FTD across refractive groups. **(A)** SFCT decreased significantly with increasing myopic severity. Pairwise comparisons showed significant differences between all groups (all *p* < 0.001), except between low myopia and moderate-to-high myopia, where the difference remained significant (*p* = 0.002). **(B)** FTD showed a corresponding increasing trend. Significant differences were observed between most groups (all *p* < 0.001), including hyperopia vs. low myopia, hyperopia vs. moderate-to-high myopia, and low myopia vs. moderate-to-high myopia. The difference between hyperopia and pre-myopia was also significant (*p* = 0.004), while the difference between pre-myopia and low myopia was not significant (*p* = 0.363). Error bars represent standard deviations or interquartile ranges.

As presented in Table 2, both SFCT and FTD differed significantly among the four refractive groups (all *p* < 0.001). A consistent trend was observed wherein SFCT decreased and FTD increased with greater myopic severity. Pairwise comparisons further revealed that SFCT was significantly different between all group pairs (all *p* < 0.001), with the exception of the comparison between low myopia and moderate-to-high myopia, which remained significant but with a higher *p*-value (*p* = 0.002). For FTD, a significant increasing trend was also confirmed. Pairwise comparisons showed significant differences between most groups (all *p* < 0.001). The difference between hyperopia and pre-myopia was also significant (*p* = 0.004). However, the difference in FTD between the pre-myopia and low myopia groups did not reach statistical significance (*p* = 0.363). These comparative relationships are further illustrated in Figure 2.

**Table 2:** Comparison of SFCT and FTD by refractive group.

Parameter	Hyperopia group	Pre-myopia group	Low myopia group	Moderate-to-high myopia group	*p*
SFCT (μm), Mean ± SD	284.51 ± 54.57	250.99 ± 52.08	229.53 ± 54.01	208.77 ± 41.71	<0.001
FTD, Median (IQR)	0.010 (0.001,0.252)	0.013 (0.008,0.033)	0.020 (0.010,0.032)	0.030 (0.014,0.050)	<0.001

### Correlation analysis between FTD and SFCT across refractive groups

**Figure 3:**
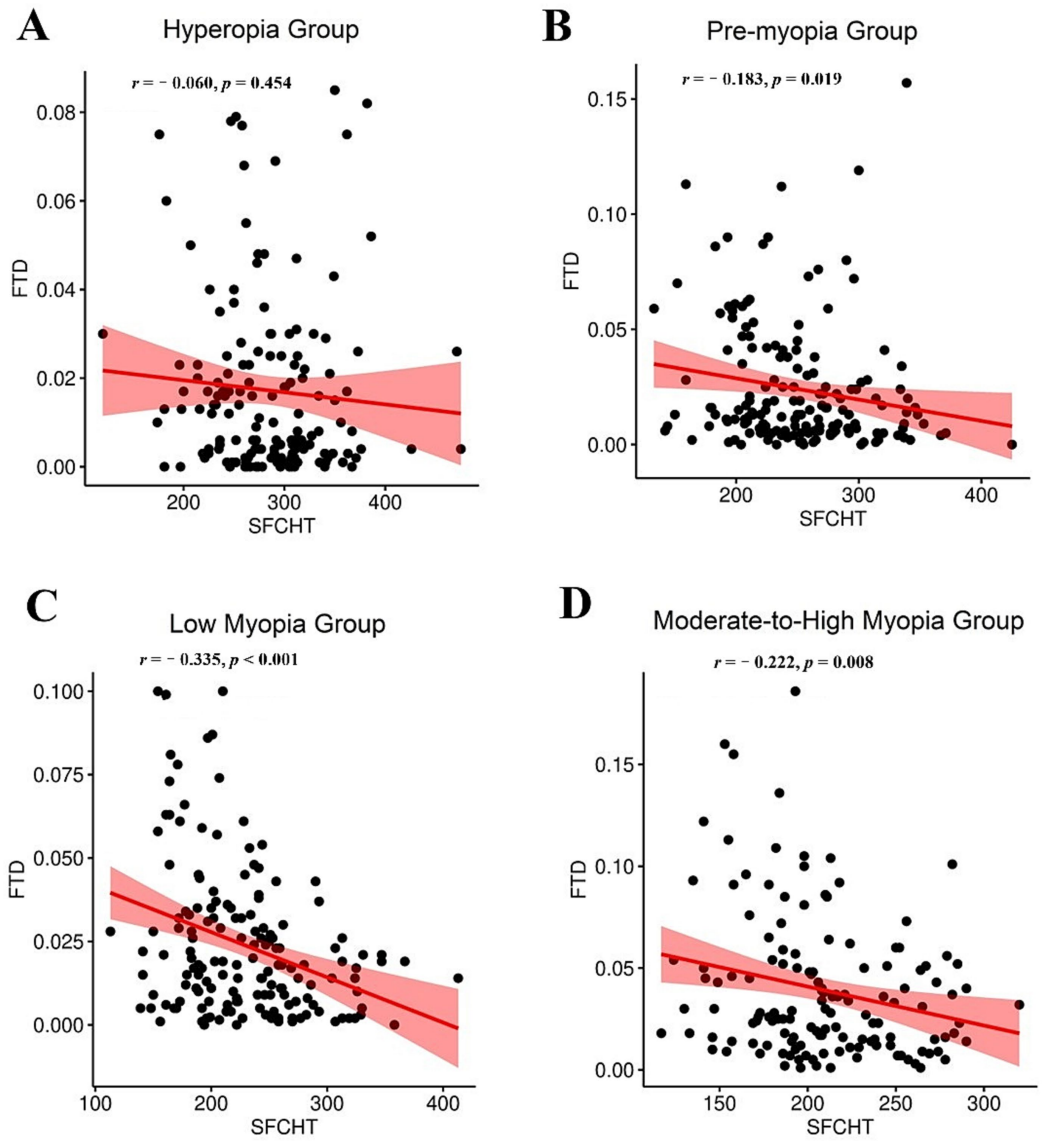
Scatterplots with trend lines illustrating the correlation between FTD and SFCT across refractive groups. **(A)** No significant correlation was found in the hyperopia group (*r* = −0.060, *p* = 0.454), **(B)** a significant negative correlation was observed in the pre-myopia (*r* = −0.183, *p* = 0.019), **(C)** low myopia (*r* = −0.335, *p* < 0.001), and **(D)** moderate-to-high myopia (*r* = −0.222, *p* = 0.008) groups. The inverse relationship between FTD and SFCT strengthened with increasing myopic severity.

Spearman correlation analysis was performed to evaluate the relationship between FTD and SFCT within each refractive group. The results are summarized in Table 3. A significant negative correlation was observed between SFCT and FTD in the pre-myopia group (*r* = −0.183, *p* = 0.019), the low myopia group (*r* = −0.335, *p* < 0.001), and the moderate-to-high myopia group (*r* = −0.222, *p* = 0.008). When all participants were considered collectively, a weak but statistically significant negative correlation was also identified (*r* = −0.283, *p* < 0.001). In contrast, no significant correlation was found between SFCT and FTD in the hyperopia group (*r* = −0.060, *p* = 0.454). To address potential confounding and clustering effects, we further applied a GEE model adjusted for age as a continuous covariate and accounting for inter-eye dependence using subject ID as the clustering variable. This comprehensive analysis confirmed the robustness of the primary findings, demonstrating that the negative correlation between FTD and SFCT remained significant after controlling for age and inter-eye dependence (*β* = −337.842, *p* < 0.001). Additionally, age-stratified analysis (6–9 years vs. 10–12 years) revealed consistently significant negative correlations in both subgroups (6–9 years: *r* = −0.361, *p* < 0.001; 10–12 years: *r* = −0.295, *p* < 0.001), further supporting the stability of the relationship across developmental stages. These correlation patterns are further illustrated in Figure 3, which presents scatterplots with trend lines for each refractive group, visually supporting the strengthening inverse relationship between SFCT and FTD with increasing myopic severity.

**Table 3:** Spearman correlation analysis between SFCT and FTD by refractive group.

SFCT	FTD	
	*r*	*p*
Hyperopia	−0.060	0.454
Pre-myopia	−0.183	0.019
Low myopia	−0.335	0.000
Moderate-to-high myopia	−0.222	0.008
Overall	−0.283	0.000

### Multivariable analysis adjusting for axial length and demographics

To assess whether the association between FTD and SFCT was independent of axial elongation and other potential confounders, we performed a GEE analysis. The model included SFCT as the dependent variable, FTD as the primary predictor, and adjusted for AL, age, and sex, with subject ID as the clustering variable to account for inter-eye correlations.

After adjustment, FTD remained significantly and inversely associated with SFCT (*β* = −240.28, 95% CI: −430.38 to −50.18, *p* = 0.013). This indicates that for each 1% increase in FTD, SFCT decreased by approximately 240.28 μm, independent of AL, age, and sex. As expected, AL was a strong independent predictor of thinner SFCT (*β* = −27.59 per mm increase, *p* < 0.001). Sex was also a significant covariate, with male subjects having a lower adjusted SFCT (*p* = 0.001), while age did not reach statistical significance in the model (*p* = 0.066). These results confirm that the FTD-SFCT relationship is not merely a surrogate of overall myopic axial elongation but represents a significant independent structural correlation.

### Sensitivity analysis using monocular data

To assess the robustness of our primary findings and address potential concerns regarding the inclusion of both eyes, we conducted a sensitivity analysis using data from only one randomly selected eye per participant (*n* = 335 eyes). The Kruskal-Wallis test confirmed that the differences in both SFCT and FTD across refractive groups remained highly significant (all *p* < 0.001), replicating the trends observed in the full dataset. Furthermore, Spearman correlation analysis on this monocular dataset continued to show a significant negative correlation between SFCT and FTD (*r* = −0.323, *p* < 0.001). The direction, magnitude, and statistical significance of these key relationships were consistent with the primary analyses that employed GEE models on the full bilateral dataset. This confirms that the core findings regarding the association between FTD and SFCT are robust to the method of handling inter-eye dependence.

### Threshold effect analysis between SFCT and FTD

**Figure 4:**
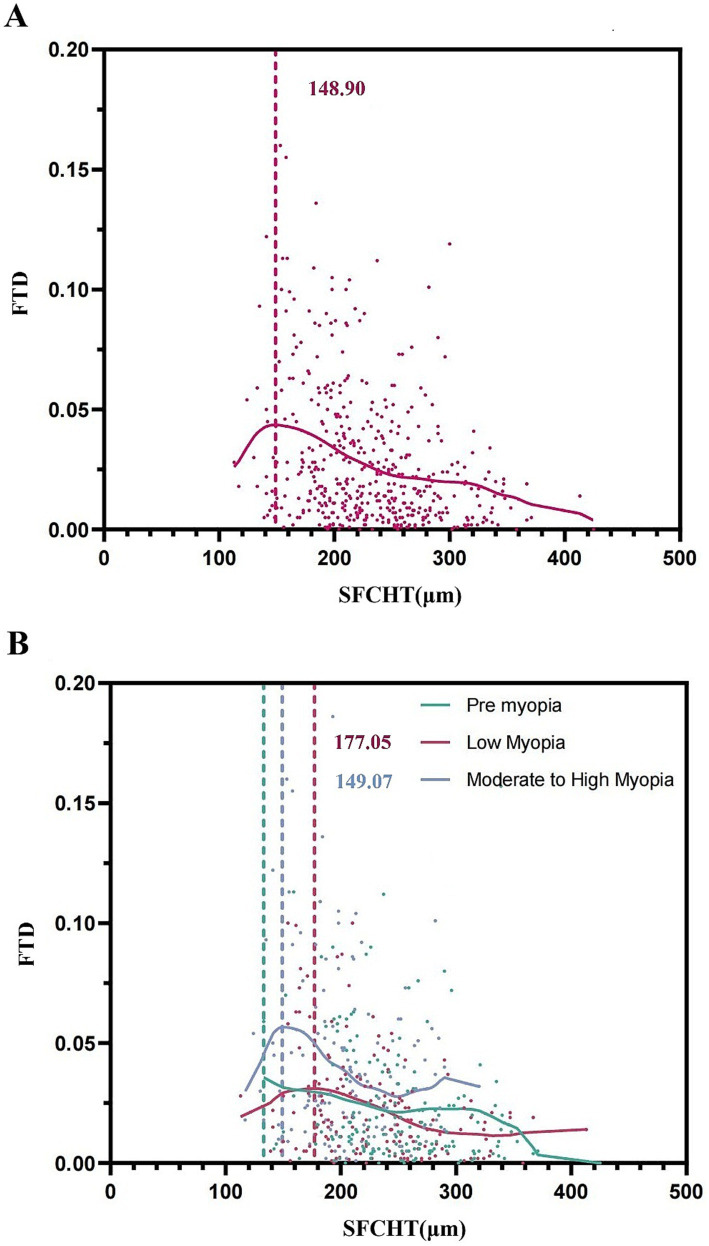
Threshold effect analysis of the relationship between SFCT and FTD using cubic spline regression and LOWESS smoothing. **(A)** Overall cohort analysis showing a significant inflection point at SFCT = 148.90 μm, beyond which FTD increased markedly with further choroidal thinning. **(B)** Refractive group-stratified analysis revealing group-specific thresholds: no significant threshold was detected in the pre-myopia group, while thresholds were identified at SFCT = 177.05 μm in the low myopia group and SFCT = 149.07 μm in the moderate-to-high myopia group. The decreasing threshold values with increasing myopic severity suggest accelerated structural changes in eyes with higher myopia.

The non-linear association between subfoveal choroidal thickness (SFCT) and fundus tessellated density (FTD) was analyzed using cubic spline regression and LOWESS smoothing, which revealed a significant threshold effect in the overall cohort wherein once SFCT decreased below 148.90 μm, the rate of increase in FTD accelerated markedly (*p* < 0.001 for slope change), as shown in Figure 3. When analyzed by refractive group, distinct thresholds emerged: no significant threshold was detected in the Pre-myopia group, whereas in the Low Myopia group, a threshold was observed at SFCT = 177.05 μm, and in the Moderate-to-High Myopia group, the threshold occurred at SFCT = 149.07 μm. These results indicate that the relationship between SFCT and FTD is nonlinear, characterized by a critical inflection point beyond which FTD increases rapidly with progressive choroidal thinning. This inflection point may serve as a potential reference marker for myopia management. Furthermore, the threshold value decreases with increasing myopic severity, indicating earlier and more pronounced structural changes in eyes with higher degrees of myopia, as illustrated in Figure 4.

### Segmented linear regression analysis

To statistically validate the non-linear threshold effect suggested by the cubic spline and LOWESS analyses, a segmented linear regression model was employed. The breakpoint was prespecified at SFCHT = 148.90 μm, as identified in the initial exploratory analysis. A derived variable, SFCHT_Excess_Pos, representing the positive excess of SFCHT beyond this breakpoint [i.e., max(0, SFCHT - 148.90)], was created.

The regression model, fitted to a subset of the data (*n* = 298), confirmed a significant change in the relationship between SFCT and FTD at the breakpoint. The model was statistically significant (*F* = 29.09, *p* < 0.001), and the SFCHT_Excess_Pos term was a significant predictor (*p* < 0.001), accounting for approximately 8.9% of the variance in FTD (R^2^ = 0.089). It is important to note that due to perfect collinearity between the original SFCHT variable and the constructed excess term in this simplified model, the precise estimation of separate slopes below and above the breakpoint was precluded. Nevertheless, the significant coefficient for the excess term provides formal statistical support for the existence of a threshold effect, indicating that the rate of increase in FTD changes significantly once SFCHT decreases below approximately 149 μm.

## Discussion

The present study demonstrates a significant nonlinear relationship between SFCT and FTD among children with varying refractive statuses, characterized by a distinct threshold effect. Specifically, we identified a critical inflection point at an SFCT of 148.90 μm in the overall cohort, beyond which further choroidal thinning was associated with a markedly accelerated increase in FTD. This finding is consistent with previous research highlighting the close anatomical and functional relationship between choroidal thickness and fundus tessellation, and further suggests that SFCT ≤148.90 μm may serve as a potential target for myopia management (21–23). The progressive decrease of this SFCT threshold from the low myopia group (177.05 μm) to the moderate-to-high myopia group (149.07 μm) suggests that eyes with more advanced myopia exhibit accelerated structural alterations at a higher baseline choroidal thickness, potentially indicating an earlier and more pronounced compromise in choroidal integrity and supportive function (7, 15, 24).

Our results corroborate existing literature confirming a moderate inverse association between SFCT and FTD, particularly in myopic eyes (21, 22, 25). The absence of a significant correlation in the hyperopia group underscores the notion that the tessellation process is intrinsically linked to myopic deformation and subsequent choroidal thinning, rather than being a mere anatomical variant (23, 26). This is consistent with large-scale studies showing a graded decrease in SFCT with increasing tessellation grade (11, 21). The tessellated fundus is a common feature in myopia, though it can also appear in emmetropic or even hyperopic eyes in children (27). The choroid plays a key role in regulating eye growth and myopia development. Choroidal thickness indirectly reflects choroidal blood supply; a reduction in choroidal blood flow can lead to scleral ischemia and hypoxia, resulting in scleral thinning and weakening (27, 28). The exact complex relationship between FTD and SFCT, however, remains to be fully elucidated.

From a broader perspective, SFCT alterations have been documented across various ocular conditions. In central serous chorioretinopathy (CSC), SFCT is significantly increased compared to healthy eyes, reflecting choroidal vascular congestion and exudation (19, 29, 30). Conversely, conditions like diabetic retinopathy show more complex patterns. This underscores that SFCT is a dynamic parameter responsive to both local ocular pathophysiology and systemic influences.

Regarding CST, measurable differences were observed across the four refractive groups; however, these variations did not exhibit a consistent gradient correlating with myopic severity. No statistically significant differences in CST were detected among the hyperopia, pre-myopia, and low myopia groups. In contrast, eyes with moderate-to-high myopia exhibited increased CST compared to the other groups. This finding aligns with previous studies indicating that, although choroidal thickness is significantly reduced across all macular subfields in eyes with tessellated fundus, CST remains relatively stable (22). These results suggest that CST changes may follow a more complex and non-linear pattern compared to choroidal thinning, with retinal thickening occurring predominantly in advanced myopia, potentially due to compensatory mechanisms or structural stretching (7, 15, 22). The absence of extreme axial elongation in our cohort further supports the notion that choroidal thinning likely precedes measurable changes in retinal thickness during the early stages of myopic progression.

The application of AI-based quantitative analysis for FTD measurement represents a significant methodological advancement over traditional qualitative grading (11, 25, 26, 31). This approach, combined with standardized EDI-OCT for SFCT assessment, enhances the objectivity and reproducibility of evaluating these intertwined biomarkers (11, 21, 22). Our use of spline regression and LOWESS smoothing effectively captured the complex nonlinear dynamics, a methodological approach gaining traction in oculomics for identifying critical transition points in disease progression (16, 29, 32).

SFCT is one of the most important biometric parameters of the eye and is directly or indirectly associated with axial ametropias and maculopathies such as myopic macular degeneration and pachychoroid-associated macular diseases, to name only a few (33–35). Notably, FTD has emerged as a promising surrogate biomarker that closely reflects choroidal thickness and myopic changes (22, 23). In the future, FTD may serve as a more accessible, low-cost, and easily detectable biomarker for widespread screening. Although SFCT can be measured relatively easily and non-invasively with high precision, its assessment requires costly ophthalmological devices and equipment, which are not readily available and are both operator-dependent and time-consuming. Therefore, there is growing interest in estimating SFCT and other correlated parameters such as FTD using alternative methods. Since fundus photographs can be acquired using widely available devices, including smartphones, we sought to explore whether such images could be utilized for estimating this biometric parameter through deep-learning algorithms. Previous studies have already demonstrated the utility of artificial intelligence in medical image analysis and disease diagnosis (36–38), supporting the feasibility of this approach.

Several limitations warrant consideration. First, the cross-sectional design precludes establishing causal inferences; longitudinal studies are needed to validate the predictive value of the identified thresholds. Second, the sample was drawn from a single clinical center, which may limit generalizability. Future research should incorporate multi-modal imaging, such as OCT angiography, to explore the microvascular underpinnings of these structural changes (39–41) and investigate whether tessellation density is associated with generalized choroidal thinning or if the three distinct layers of the choroid (choriocapillaris, Haller’s layer, Sattler’s layer) are differentially affected in eyes with tessellated fundus (26). Additionally, further investigation into systemic associations could provide a more comprehensive understanding of the implications of choroidal thinning.

In conclusion, this study provides robust evidence for a nonlinear relationship between SFCT and FTD in children, characterized by a defined threshold effect that varies with myopic severity. The integration of AI-driven FTD quantification and advanced statistical modeling offers a refined framework for understanding chorioretinal changes in myopia. These findings underscore the value of combining structural biomarkers for improving early detection, monitoring, and potentially guiding future sight-preserving interventions in pediatric myopia.

## Data Availability

The raw data supporting the conclusions of this article will be made available by the authors, without undue reservation.
